# Extracellular Vesicles Act as Nano-Transporters of Tyrosine Kinase Inhibitors to Revert Iodine Avidity in Thyroid Cancer

**DOI:** 10.3390/pharmaceutics13020248

**Published:** 2021-02-10

**Authors:** Ramya Lakshmi Rajendran, Sanjita Paudel, Prakash Gangadaran, Ji Min Oh, Eun Jung Oh, Chae Moon Hong, Sangkyu Lee, Ho Yun Chung, Jaetae Lee, Byeong-Cheol Ahn

**Affiliations:** 1 Department of Nuclear Medicine, School of Medicine, Kyungpook National University, Daegu 41944, Korea; ramyag@knu.ac.kr (R.L.R.); prakashg@knu.ac.kr (P.G.); ojm0366@naver.com (J.M.O.); jaetae@knu.ac.kr (J.L.); 2 BK21 FOUR Community-Based Intelligent Novel Drug Discovery Education Unit, College of Pharmacy and Research Institute of Pharmaceutical Sciences, Kyungpook National University, Daegu 41566, Korea; sanjitapdl99@gmail.com (S.P.); pharm239@gmail.com (S.L.); 3 BK21 FOUR KNU Convergence Educational Program of Biomedical Sciences for Creative Future Talents, School of Medicine, Kyungpook National University, Daegu 41944, Korea; hy-chung@knu.ac.kr; 4 Department of Plastic and Reconstructive Surgery, CMRI, School of Medicine, Kyungpook National University, Kyungpook National University Hospital, Daegu 41944, Korea; fullrest74@hanmail.net; 5 Department of Nuclear Medicine, Kyungpook National University Hospital, Daegu 41404, Korea; shahking@hanmail.net

**Keywords:** extracellular vesicles, tyrosine kinase inhibitor, drug delivery, radioactive iodine, thyroid cancer

## Abstract

A new approach for using extracellular vesicles (EVs) to deliver tyrosine kinase inhibitors (TKIs) to enhance iodine avidity in radioactive iodine-refractory thyroid cancer is needed. We isolated and characterized primary human adipose-derived stem cells (ADSCs) and isolated their EVs. The EVs were characterized by transmission electron microscopy, nanoparticle tracking analysis, and western blotting. A new TKI was loaded into the EVs by incubation (37 °C; 10 min) or sonication (18 cycles; 4 s per cycle) with 2 s intervals and a 2 min ice bath every six cycles. TKI loading was confirmed and measured by mass spectrometry. EV uptake into radioactive iodine-refractory thyroid cancer cells (SW1736 cells) was confirmed by microscopy. We treated the SW1736 cells with vehicle, TKI, or TKI-loaded EVs (sonication TKI-loaded EVs [EVs^TKI(S)^]) and examined the expression of iodide-metabolizing proteins and radioiodine uptake in the SW1736 cells. ADSCs cells showed >99% of typical stem cell markers, such as CD90 and CD105. The EVs displayed a round morphology, had an average size of 211.4 ± 3.83 nm, and were positive for CD81 and Alix and negative for cytochrome c. The mass spectrometry results indicate that the sonication method loaded ~4 times more of the TKI than did the incubation method. The EVs^TKI(S)^ were used for further experiments. Higher expression levels of iodide-metabolizing mRNA and proteins in the EVs^TKI(S)^-treated SW1736 cells than in TKI-treated SW1736 cells were confirmed. EVs^TKI(S)^ treatment enhanced ^125^I uptake in the recipient SW1736 cells compared with free-TKI treatment. This is the first study that demonstrated successful delivery of a TKI to radioactive iodine-refractory thyroid cancer cells using EVs as the delivery vehicle. This approach can revert radioiodine-resistant thyroid cancer cells back to radioiodine-sensitive thyroid cancer cells.

## 1. Introduction

Anaplastic thyroid cancer (ATC) is the most aggressive type of thyroid tumor, and it has a high mortality rate because of the high rate of genetic modifications in tumor cells [1]. ATC has poor prognosis, primarily because it is usually diagnosed in the late and advanced stages of tumor progression and disease. Therefore, curative resection of the tumor is not possible for most ATC patients [2,3]. The sodium iodide symporter (NIS) is a transmembrane glycoprotein with 13 transmembrane domains that mediates the active transport of both sodium and iodide together from the extracellular fluid [4].

Extracellular vesicles (EVs) are nanovesicles made of a lipid bilayer, and are released from most types of cells. EVs are classified as exosomes, microvesicles, and apoptotic bodies based on their biogenesis and release pathways. EVs deliver proteins, lipids, miRNA, mRNA, and even DNA to recipient cells [5,6,7]. Hence, research scientists are concentrating on exploiting EVs as a drug delivery nano-transporter. This drug delivery nano-transporter is slowly and steadily gaining attention because it has shown potential for improving the targeted delivery of drugs, which is a challenge faced by conventional drugs [8,9].

A number of studies have reported benefits from using EVs as a drug delivery nano-transporter in preclinical models [10,11,12]. Therefore, EVs are evolving as a potential candidate for a novel drug delivery nano-transporter. A few human clinical trials using EVs for cancer treatment have already been completed, while some are ongoing [13,14,15], and encouraging results regarding the feasibility and safety of EVs have been reported.

Numerous studies using EVs from mouse or human cells have isolated cell lines for loading and delivering drugs, including EL-4 (mouse lymphoma cell line), RAW264.7 (mouse macrophage cell line) [16], LNCaP and PC-3 (prostate cancer cell lines) [17], and U937 cells (monocyte cell line) [18], as well as other MSCs (human bone marrow-derived mesenchymal stem cell lines) [19]. However, studies that isolate and characterize drug loading and delivery in primary human ADSCs are very few in number [20]. Herein, we reported a methodical approach for the isolation, enrichment, and characterization of EVs from primary human ADSC-derived EVs for use in drug delivery nano-transporters. We chose primary cells over other cell lines because there are misidentified and contaminated cell lines. Most cell lines differ genetically and phenotypically from their tissue origin every so often [21,22]. These genetical and phenotypical alterations are reflected in EVs, as they are derived from and secreted by cells.

Here, we hypothesized that the EV-mediated delivery of a TKI to ATC cells (SW1736 cells) might enhance the therapeutic effects of the TKI on the ATC cells. We isolated and characterized human primary adipose tissue-derived stem cells (ADSCs). The EVs of the ADSCs were isolated and characterized. We used a newly identified TKI, 5-(5-{4H,5H,6H-cyclopenta [b]thiophen-2-yl}-1,3,4-oxadiazol-2-yl)-1-methyl-1,2-dihydropyridin-2-one, which restores endogenous NIS expression in ATC cells. We investigated two different drug loading methods (sonication and incubation) to load the TKI into the EVs. Furthermore, we investigated the enhancement of radioactive iodine (RAI) avidity from EV-mediated TKI delivery in vitro.

## 2. Materials and Methods

### 2.1. Ethics Approval

This study was approved by the Institutional Review Board (IRB) of Kyungpook National University Hospital (IRB no. 2020-02-035-002) and performed in accordance with the principles of the Declaration of Helsinki.

### 2.2. Collection of ADSC

Adipose tissues obtained from discarded subcutaneous tissue during flap surgery were washed with equal volumes of phosphate-buffered saline (PBS). Next, enzymatic degradation was performed at 37 °C using 0.075% collagenase type I (Worthington Biochemical, Lakewood, NJ, USA). To remove the debris from the connective tissues, the degraded tissue was filtered, and the supernatant of the adipocyte layer was separated. The cell suspension was centrifuged at 200× *g* for 10 min. Contaminated erythrocytes were removed by adding erythrocyte lysis buffer (Sigma-Aldrich, St. Louis, MO, USA) at pH 7.3. The stromal cells were rinsed twice with PBS, and the ADSCs were collected [23].

### 2.3. Cell Culture

The ADSCs were cultured in Dulbecco’s Modified Eagle’s medium (Gibco, Carlsbad, CA, USA) supplemented with 10% fetal bovine serum (FBS) (Hyclone, Logan, UT, USA). SW1736 cells, which are a human ATC cell line, were received as a gift from Dr. Minho Shong (School of Medicine, Chungnam National University). These cells were cultured in RPMI-1640 medium (Hyclone). Both the ADSCs and SW1736 cells were supplemented with 10% EV-depleted FBS (FBS underwent ultracentrifugation at 120,000× *g* and 4 °C for 18 h) and 1% penicillin and streptomycin in a CO_2_ incubator at 37 °C.

### 2.4. Flow Cytometry Analysis

The ADSCs (5 × 10^4^) were incubated with antibodies against CD90 and CD105 (BD Bioscience, Inc., San Jose, CA, USA). Ten thousand fluorescent-tagged cells were captured using a MoFlo XDP automated high-speed cell sorter (Beckman Coulter, Brea, CA, USA), and analyzed using FlowJo 10.0 software (FlowJo, Inc., Fort Collins, CO, USA). The cell population was expressed as a percentage.

### 2.5. Isolation of ADSC EVs

The ADSCs culture media was collected, and the EVs were isolated from them, as illustrated in Figure S1. The supernatant was centrifuged at 1500× *g* for 10 min to remove the cells. Next, it was centrifuged again at 4000× *g* for 20 min to remove the cell debris. The collected supernatant was filtered through a 0.45 µm syringe filter. The samples underwent ultracentrifugation at 100,000× *g* for 60 min. The collected pellets were washed with PBS and then underwent ultracentrifugation at 100,000× *g* for 60 min. The pellets were reconstituted in 50–100 µL PBS and then stored at −80 °C until use. All ultracentrifugation procedures were performed at 4 °C using a SW28 rotor (Beckman Coulter, Brea, CA, USA). The EV concentration was measured with a Pierce BCA protein assay kit (Thermo Fisher Scientific, Waltham, MA, USA).

### 2.6. Western Blot Analysis

Western blot analysis was performed as described in a previous study [24]. Whole cells and EV lysates were prepared in RIPA buffer (Thermo Fisher Scientific, Waltham, MA, USA). Equal amounts of protein were loaded and separated using 10% sodium dodecyl sulfate-polyacrylamide gel electrophoresis (SDS-PAGE). The proteins were transferred to PVDF membranes (Millipore, Burlington, MA, USA). The blots were first probed with primary antibodies (1:2500 CD81, Abcam; 1:2500 Alix, Abcam; 1:5000 cytochrome c, Abcam). Next, they were probed with secondary antibodies conjugated with horseradish peroxidase. The signals were detected using enhanced chemiluminescence (GE Healthcare, Chicago, IL, USA) according to the manufacturer’s instructions. Band intensity was measured by GelQuant.NET software (version 1.8.2) (BiochemLabSolutions.com, San Francisco, CA, USA).

### 2.7. Nanoparticle Tracking Analysis (NTA)

The size of the ADSC EVs was measured by NTA using a NanoSight LM10 (Malver) with a 640 nm laser according to the described protocol. The ADSC EVs were diluted in milli-Q water (1:1000), the sample was injected into the chamber through a sterile syringe, and the measurements were performed in triplicates. Next, these results were analyzed [25].

### 2.8. Transmission Electron Microscopy (TEM)

The samples were resuspended in 100 µL of 2% paraformaldehyde. The EV pellets were attached to Formvar/carbon-coated electron microscope (EM) grids, and to avoid exposing the samples to light, dryness, or other damage, they were covered with aluminum foil for 20 min. The samples were washed with 100 µL PBS and incubated in 1% glutaraldehyde for 5 min. The samples were washed with distilled water seven times (2 min each), and then they were viewed under an HT 7700 transmission EM to measure the size of the EVs [26].

### 2.9. TKI Loading into the EVs

The TKI used in this study, 5-(5-{4H,5H,6H-cyclopenta [b]thiophen-2-yl}-1,3,4-oxadiazol-2-yl)-1-methyl-1,2-dihydropyridin-2-one, was purchased from Enamine (Monmouth Junction, South Brunswick, NJ, USA). The TKI was dissolved in dimethyl sulfoxide (DMSO) and stored at −20 °C for further use. TKI-loaded EVs and EVs alone were sonicated (500 V, 2 kHz, 20% power; six cycles with 4 s pulse/2 s pause), and then cooled on ice for 2 min. Next, these steps were repeated two more times. A separate batch of TKI-loaded EVs and EVs alone were incubated at 37 °C in a shaking incubator for 10 min. After sonication and incubation were performed, the samples underwent gradient ultracentrifugation at 120,000× *g* and 4 °C for 3 h. The TKI-loaded EVs that were loaded by sonication (EVs^TKI(S)^) and the TKI-loaded EVs that were loaded by incubation (EVs^TKI(I)^) were collected from the interface of 60% and 20% iodixanol layers, respectively.

### 2.10. Liquid Chromatography–Mass Spectrometry (LC-MS) Analysis Sample Preparation

The blank EVs, EVs^TKI(S)^, and EVs^TKI(I)^ were prepared for LC-MS analysis using the following procedure. 5 µL of each sample was combined with 95 µL of PBS. Next, 900 µL of 100% ACN with 0.1% formic acid and 0.25 µM reserpine was added to each sample. Next, the samples underwent ultrasonication with an ultrasonic homogenizer (Sonics Vibra Cell, Newtown, CT, USA) with 30% amplitude (5 s/pulse for each cycle) for protein lysis. The samples were centrifuged at 13,000 rpm and 4 °C for 10 min, and the resulting supernatant was analyzed by LC-MS.

### 2.11. TSQ Triple Quadrupole Mass Spectrometry

To quantify the sample concentration, mass spectrometry analysis was performed using a TSQ Vantage triple quadrupole mass spectrometer consisting of a HESI-II spray source coupled to a Vanquish UHPLC system (Thermo Fisher Scientific, Waltham, MA, USA). An ACE 5 C18 column (50 × 2.1 mm) was used to separate the analytes in the samples. The mobile phase contained 100% water with 0.1% formic acid (mobile phase A) and acetonitrile with 0.1% formic acid (mobile phase B), and had a flow rate of 0.40 mL/min at 40 °C. The gradient conditions were as follows: 20% B for 0–0.05 min, which was gradually increased from 20% to 95% B at 0.05–1 min; 95% B at 1–3.20 min; 95–20% B at 3.20–3.50 min; and 20% B at 3.50–5.5 min. The MS operating conditions were as follows: electrospray ionization in negative mode; 3000 V and 4000 V in positive mode; capillary temperature, 350 °C; vaporizer temperature, 300 °C; sheath gas pressure, 35 Arb; auxiliary gas pressure, 10 Arb. For the LCMS-MS analysis, 2 μL of each of sample was injected. Finally, all data were analyzed using Xcalibur software (Thermo Fisher Scientific, Waltham, MA, USA).

### 2.12. LC-MS Method Validation

TKI stock solutions were prepared in blank EVs to generate a calibration curve and linearity assessment. The concentrations of TKI in the calibration curve were 1, 5, 10, 20, 50, 100, 200, 500, 1000, 2000, 5000, 10,000, and 20,000 ng/mL. The peak area ratios of the analytes/internal standard (IS) and sample concentrations were used to prepare the calibration curves. Five concentrations (10, 100, 1000, 5000, and 20,000 ng/mL) were chosen for inter-day and intra-day validation.

To evaluate the accuracy and precision (RSD) of the TKI measurements, blank EVs were spiked with known concentrations of TKI or QC samples. The concentrations used were 10, 100, 1000, 5000, and 20,000 ng/mL (*n* = 5). Moreover, the accuracy and RSD of intra-day and inter-day measurements for each concentration were analyzed after five consecutive days and on each individual day, respectively.

### 2.13. EV Interaction and Internalization Assay

The interaction and internalization of the EVs, EVs^(S)^, EVs^(I)^, EVs^TKI(S)^, and EVs^TKI(I)^ were analyzed using confocal microscopy. The EVs were labeled with lipophilic dye (DiD) by incubation at 37 °C for 20 min, and subsequently washed with PBS by ultracentrifugation, according to the procedure described above. SW1736 cells were incubated at 37 °C with the DiD-labeled EVs (5 µg) for 2 h before undergoing methanol fixation. Antifade agent was used to mount the coverslips (Vector Laboratories, Burlingame, CA, USA). EV uptake was observed using an LSM 800 laser scanning microscope (Carl Zeiss, Baden-Württemberg, Germany).

### 2.14. In Vitro Treatment

SW1738 cells were treated with DMSO (control), TKI (0.5 µg/mL), or EVs^TKI(S)^ (0.5 µg/mL). Next, the cells were supplemented with 10% EV-depleted FBS and 1% penicillin and streptomycin at 37 °C in a CO_2_ incubator for 72 h. The cell pellets were collected and analyzed by real-time polymerase chain reaction (PCR) and western blotting.

### 2.15. Real-Time PCR

All reactions included SsoAdvancedTM Universal SYBR Green Supermix (Bio-Rad, Hercules, CA, USA), 50 ng cDNA, and 10 µM primers. Amplification was performed under the following cycle conditions: 95 °C for 10 min, followed by 40 cycles at 95 °C for 15 s and at 60 °C for 60 s in an ABI-7500 detection system (Applied Biosystems, Foster, CA, USA), according to the manufacturer’s instructions. Differences between the samples and controls were calculated using the 2^−ΔΔCt^ method, as previously described [27].

### 2.16. In Vitro ^125^I Uptake Assay

SW1738 cells (5 × 10^4^) were seeded into 24-well plates and incubated with DMSO (control), TKI (0.5 µg/mL), or EVs^TKI(S)^ at 5% CO_2_ and 37 °C in a CO_2_ incubator for 72 h. After the samples underwent incubation, the medium was aspirated, and the SW1738 cells were washed with warm Hank’s balanced salt solution (HBSS) containing 0.5% BSA (bHBSS). The cells were subsequently incubated with 500 µL bHBSS, 37 kBq carrier-free ^125^I (Perkin-Elmer, Waltham, MA, USA), and 100 µmol/L sodium iodide (NaI) (specific activity: 740 MBq/mM) at 37 °C in a humidified incubator for 30 min. To prevent ^125^I uptake, the SW1738 cells were treated with 50 µM potassium perchlorate (KClO_4_), which is a competitive inhibitor of NIS, for 30 min before adding ^125^I. After undergoing incubation, the cells were washed twice with chilled bHBSS and lysed with 500 µL of RIPA buffer. Radioactivity was measured using a Cobra II gamma counter (Canberra Packard, Mississauga, ON, Canada). Uptake values were normalized to the total protein concentration, as determined by a BCA protein assay kit. The results are expressed as counts per min/µg.

### 2.17. Statistical Analysis

All data are expressed as the mean ± standard deviation (SD). Two-group comparisons were statistically analyzed by Student’s *t*-test in Microsoft Excel (Microsoft, Redmond, WA, USA) or GraphPad Prism 9 software version 9.0.0(121) (GraphPad Software, San Diego, Inc., CA, USA). A *p*-value < 0.05 was considered statistically significant.

## 3. Results

### 3.1. Collection and Characterization of ADSCs

**Figure 1:**
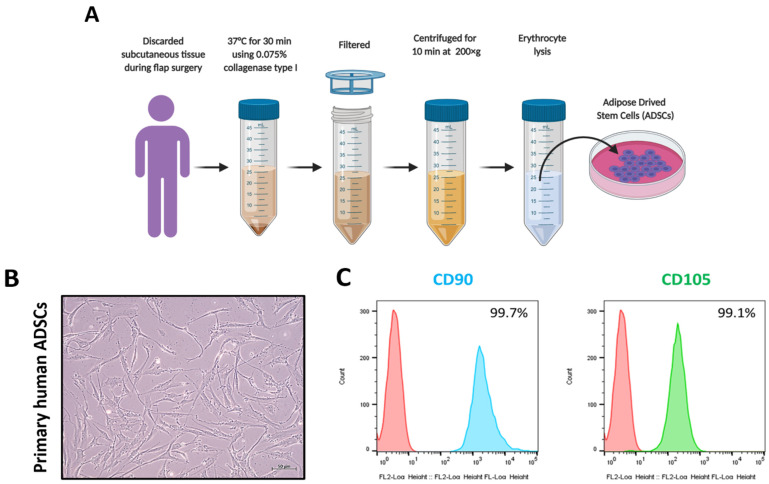
Isolation and Characterization of human primary ADSCs. (**A**) Schematic diagram, as created with BioRender.com software, of the process used for isolating the human primary ADSCs. (**B**) Phase-contrast imaging of the human primary ADSCs (scale bar: 50 µm). (**C**) Flow cytometry analysis of CD90 and CD105 in human primary ADSCs.

ADSCs were isolated from adipose tissue derived from subcutaneous tissue discarded during flap surgery, as illustrated in Figure 1A. The morphology of the isolated primary ADSCs was observed under an inverted microscope. The ADSCs displayed bipolarity or multipolarity with an elongated shape or fibroblast-like morphology. No differences in morphology were observed in the cells from different passages (up to five passages of cells were used in this study) (Figure 1B). CD90 and CD105 stem cell surface markers were confirmed by flow cytometry in the fifth passage of ADSCs. The percentages of ADSCs expressing the CD90 and CD105 cell surface markers were 99.7% and 99.1%, respectively (Figure 1C). These results confirm that the isolated cells were indeed the ADSCs.

### 3.2. Characterization of EVs

**Figure 2:**
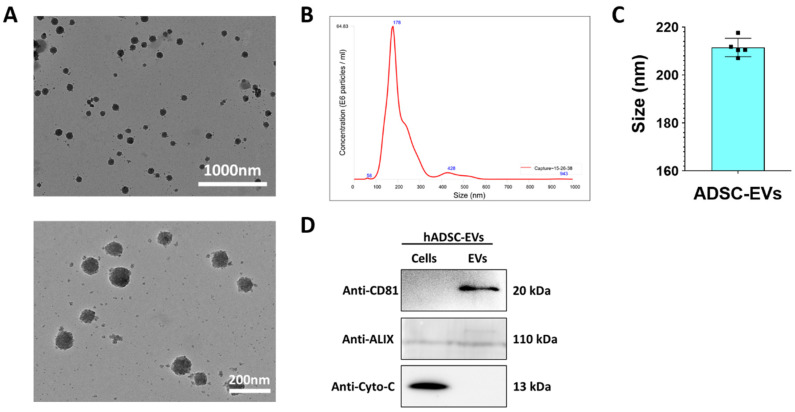
Characterization of the ADSC EVs. (**A**) Morphology of human primary ADSC EVs confirmed by TEM (scale bar: 1000 and 200 nm). (**B**,**C**) NTA of the ADSC EVs (*n* = 5). The results are expressed as the mean ± SD. (**D**) Western blot analysis of the ADSCs and ADSC EVs, for which anti-CD81, anti-ALIX, and anti-cytochrome c antibodies were used.

EVs were isolated from the cultured media of primary ADSCs by ultracentrifugation, as illustrated in Figure S1. The EVs were characterized using TEM, NTA, and western blotting. The morphology of the EVs was observed by TEM, which revealed the typical round or spherical morphology of EVs and indicated that most of the EVs were intact (upper and lower panels; Figure 2A). The size of the EVs was confirmed by NTA, which displayed a single enriched peak at 178 nm and indicated that the average diameter of the EVs was 211.4 ± 3.83 nm (Figure 2B,C). The enrichment and purity of the EVs were confirmed by well-characterized positive and negative markers of EVs. Western blot results showed enrichment of CD81 (a membrane protein) and Alix (a cytoplasmic protein) in the EV fraction. The negative marker cytochrome c (a mitochondrial protein) was present only in the cells (Figure 2D). These results confirmed that the EVs isolated from ADSCs were intact and without contamination of cells.

### 3.3. LC-MS Method Validation

There was no interfering peak in the blank EVs. Therefore, the peak retention time of TKI and IS did not interfere with the blank EVs. The calibration curve equation, y = 0.006x + 0.0358 [R^2^ = 0.9999], which was used to quantify the TKI in the TKI-loaded EV sample, was linear. The lower limit of detection and lower limit of quantitation for the TKI concentrations were 5 and 10 ng/mL, respectively. The within-day and between-day precision (RSD%) calculations of the TKI concentrations were <5.1% and <5.2%, respectively, whereas the within-day and between-day accuracy calculations of the TKI concentrations were <114.8% and <114.4%, respectively (Supplemental Table S1). These results indicate that the precision and accuracy calculations are within the acceptable ranges and that the method is valid.

### 3.4. TKI Loading into EVs

**Figure 3:**
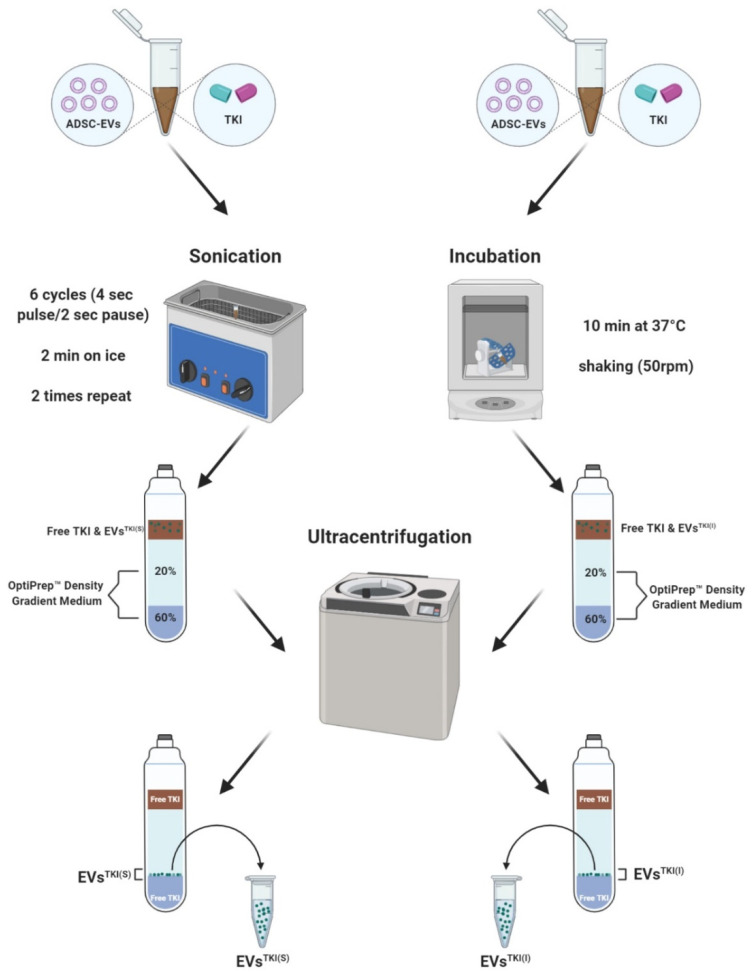
Illustration of the process used to load the TKI into the EVs. This illustration was created with BioRender.com software.

**Figure 4:**
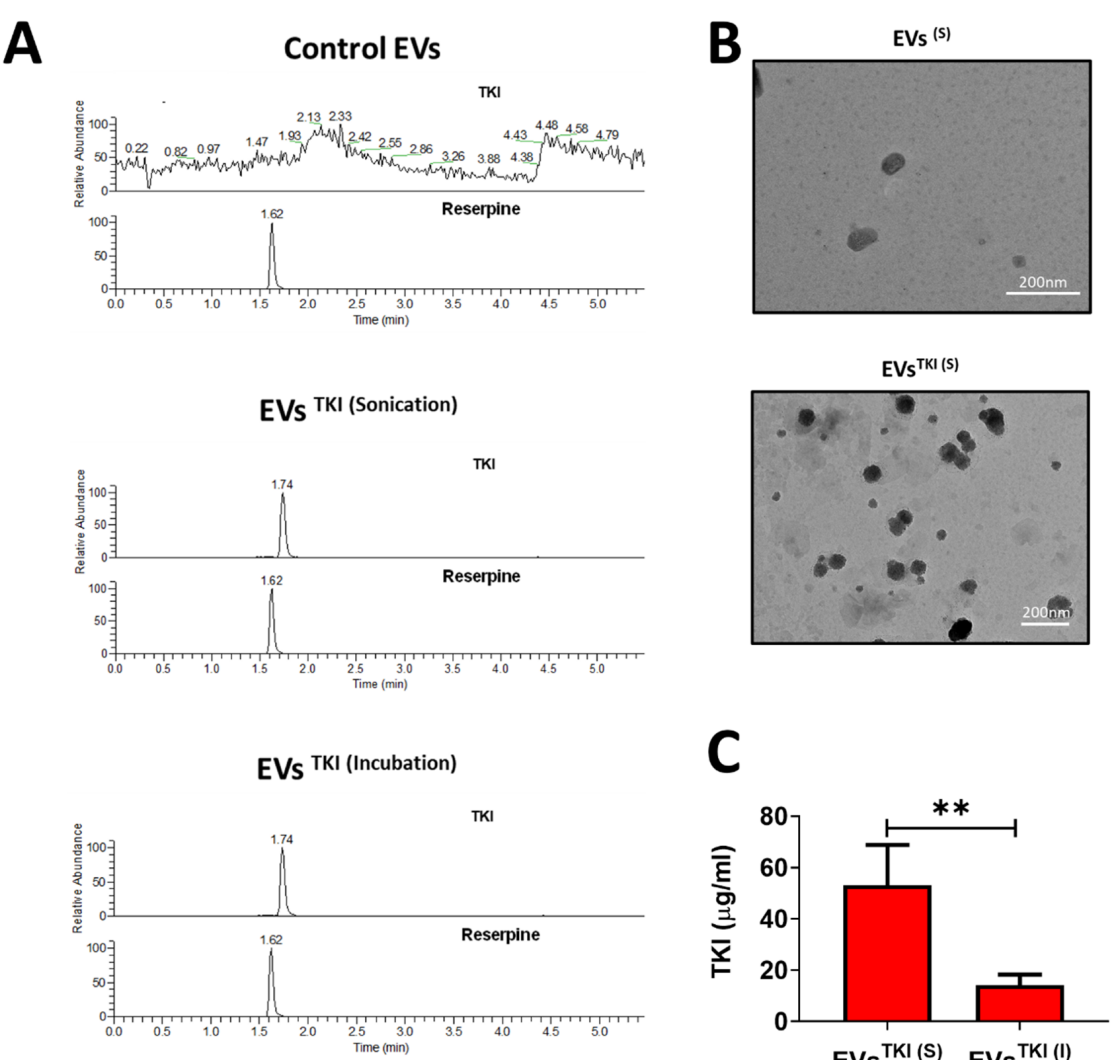
TKI loading into the EVs. (**A**) A representative graphical image of the mass spectrometry chromatogram for the TKI in the EVs, EVs^TKI(S)^, and EVs^TKI(I)^. (**B**) Quantifications of TKI in EVs^TKI(S)^ and EVs^TKI(I)^ (*n* = 3). The results are expressed as mean ± SD. (**C**) Morphology of the EVs^(S)^ and EVs^TKI(S)^ confirmed by TEM (scale bar: 200 nm). ** *p* < 0.01 (by Student’s *t*-test).

A newly identified TKI that exhibits an ability to augment iodine uptake in ATC was used in the current study. Before the TKI was loaded into the EVs, we first confirmed the detection of TKI by mass spectrometry analysis. The results of the LC-MS analysis exhibited a single peak at 1.7 min for the TKI, and reserpine was used as a positive control chemical, which was also detected and had a single peak, at 1.6 min, in positive ionization mode. The representative multiple reaction monitoring mode (MRM) transitions for TKI and reserpine (IS) were 300.1–157.1 and 609.8–195.0, respectively. The representative MRM chromatograms for TKI and reserpine are shown in Figure S2. TKI was loaded into the EVs by two methods: sonication and incubation, as illustrated in Figure 3. In essence, TKI was mixed with EVs and then either sonicated or incubated. After purification of the EVs^TKI(S)^ and EVs^TKI(I)^ was performed, these two samples of TKI-loaded EVs, along with EVs alone, underwent MS analysis. The MS analysis results revealed that EVs alone did not show the exact TKI peak (1.5–2.0 min). Instead of a single peak, it showed multiple background peaks. In contrast, the EVs^TKI(S)^ and EVs^TKI(I)^ both showed a single peak at the exact same spot as the free TKI (Figure 4A). Further quantification analysis revealed that the sonication method loaded a significantly higher concentration of TKIs (*p* < 0.01), more than five times that of TKIs loaded by the incubation method (Figure 4B). The encapsulation efficiency of TKI was 0.531 ± 0.16% (sonication) and 0.141 ± 0.04% (incubation). We analyzed whether the sonication and incubation methods changed the morphology of the EVs (with or without TKI), and the results indicate that both methods did not affect the morphology of the EVs (Figure 4C and Figure S3A). These results revealed that the newly identified TKIs were detected by MS analysis, the sonication method loaded more TKIs into the EVs than did the incubation method, and the morphology was not changed by either of the two loading procedures.

### 3.5. Internalization/Uptake of EVs^TKI(S)^ and EVs^TKI(I)^ Into Human ATC Cells.

**Figure 5:**
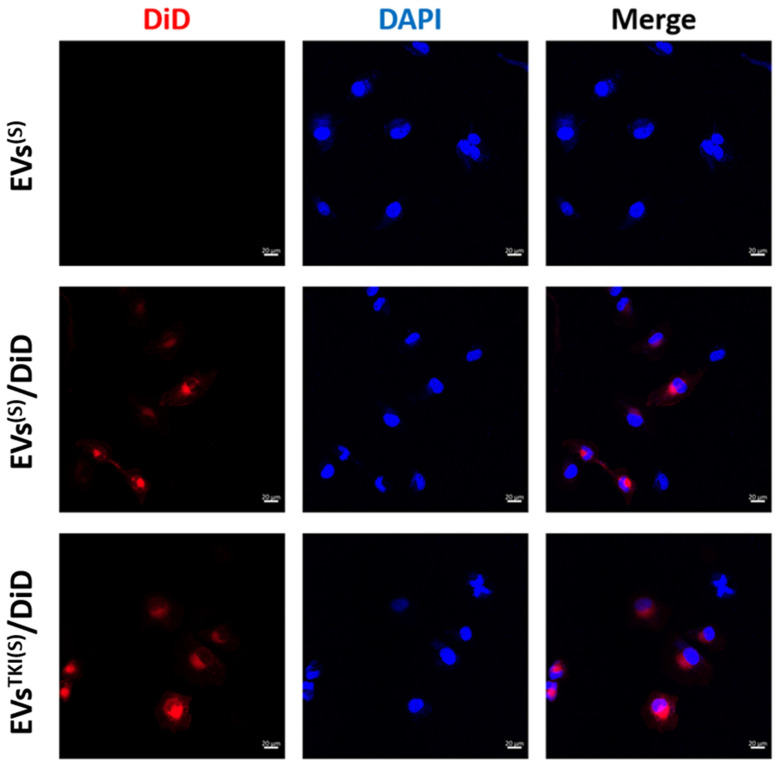
Internalization of the EVs into ATC cells. SW1736 ATC cells were incubated with EVs^(S)^, EVs^(S)^/DiD, or EVs^TKI(S)^/DiD (scale bar: 20 µm).

Internalization of the EVs into ATC cells (SW1736 cell line) was confirmed by fluorescent microscopy. The results revealed that EVs, EVs^TKI(S)^, and EVs^TKI(I)^ were actively internalized into the SW1736 cells (Figure 5 and Figure S3B).

### 3.6. Delivery of TKI by EVs Strongly Induces Expression of Thyroid-Specific Genes and Thyroid-Specific Transcription Factors in ATC Cells

**Figure 6:**
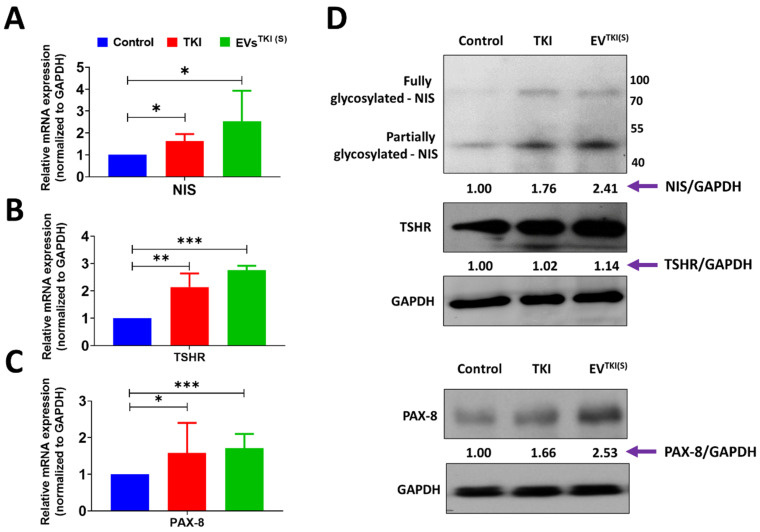
Enhancement of mRNA and protein expression levels in ATC cells after EVs^TKI(S)^ treatment. The SW1736 cells were exposed to DMSO (0.5 µg/mL), TKI (0.5 µg/mL), and EVs^TKI(S)^ (0.5 µg/mL) for 72 h. (**A**–**C**) mRNA expression levels of NIS, TSHR, and PAX-8 in ATC cells; GAPDH was used as a housekeeping gene (*n* = 3). Data are expressed as mean ± SD. (**D**) Western blot analysis of NIS, TSHR, and Pax-8 levels in ATC cells. GAPDH was used as the loading control. Band intensity was measured using GelQuant.NET software (version 1.8.2) (BiochemLabSolutions.com, CA, USA). *** *p* < 0.001, ** *p* < 0.01, * *p* < 0.05 (by Student’s *t*-test). TSHR: TSH receptor; PAX-8: paired box-8.

As TKI loading was shown to be lower in the EVs^TKI(I)^ than in the EVs^TKI(S)^, the EVs^TKI(S)^ were used in further experiments. We analyzed the mRNA expression levels of *NIS, TSHR*, and PAX-8 after treatment of DMSO (control), TKI (0.5 µg/mL), and EVs^TKI(S)^ (0.5 µg/mL; same concentration as the free TKI). The TKI and EVs^TKI(S)^ treatments increased the *NIS* mRNA expression level significantly more compared with the control (Control vs. TKI, *p* < 0.05; Control vs. EVs^TKI(S)^, *p* < 0.05). The EVs^TKI(S)^ treatment produced a substantially higher *NIS* mRNA expression level compared with the TKI treatment (Figure 6A). The TKI and EVs^TKI(S)^ treatments increased the *TSHR* mRNA expression level significantly more compared with the control (Control vs. TKI, *p* < 0.01; Control vs. EVs^TKI(S)^, *p* < 0.001). The cells treated with EVs^TKI(S)^ showed a substantially higher expression level of *TSHR* mRNA than those treated with TKI (Figure 6B). The TKI and EVs^TKI(S)^ treatments increased the PAX-8 mRNA expression level significantly more compared with the control (Control vs. TKI, *p* < 0.05; Control vs. EVs^TKI(S)^, *p* < 0.001) (Figure 6C). These results indicate that the TKI delivered by the EVs increased the expression levels of thyroid-specific genes and thyroid-specific transcription factors in ATC cells significantly more compared with the free form of TKI.

### 3.7. Delivery of TKI by an EV-Based Nano-Transporter Increases Thyroid-Specific Protein Levels in ATC Cells

The delivery of TKI by EVs increased the mRNA expression levels of *NIS, TSHR*, and PAX-8 in ATC cells. We further tested the translation levels of these genes via quantification of the proteins by western blot. The western blot results indicate that the TKI treatment increased the NIS protein level more compared with the control by a factor of 1.76, and the EVs^TKI(S)^ treatment increased the NIS protein level more compared with the control by a factor of 2.41. The TKI treatment showed negligible levels of the TSHR protein, and the EVs^TKI(S)^ treatment increased the TSHR protein level more compared with the control by a factor of 1.14. Finally, the TKI treatment increased the PAX-8 protein level more compared with the control by a factor of 1.66, and the EVs^TKI(S)^ treatment increased the PAX-8 protein level more compared with the control by a factor of 2.53 (Figure 6D). These results suggest that the delivery of TKI by EVs significantly increases the levels of thyroid-specific proteins and transcription factors in ATC cells.

### 3.8. Evaluation of NIS Function after Delivery of TKI by EVs via Assessment of RAI Accumulation

**Figure 7:**
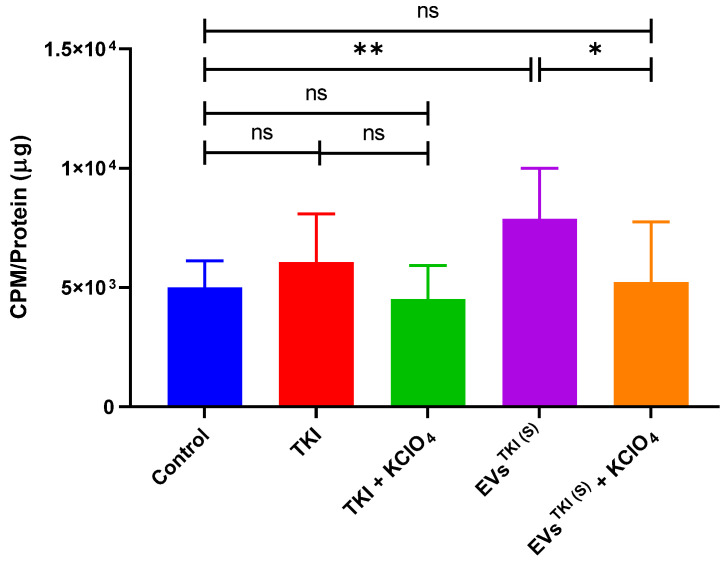
^125^I accumulation in ATC cells after treatment with TKI or EVs^TKI(S)^. The SW1736 cells were exposed to TKI (0.5 µg/mL) or EVs^TKI(S)^ (0.5 µg/mL) for 72 h and were subsequently incubated with 37 kBq carrier-free ^125^I and 10 µm/L sodium iodide at 37 °C for 30 min. ^125^I uptake after the TKI treatment and that after the EVs^TKI(S)^ treatment were compared with the ^125^I uptake after the control treatment (DMSO). KClO_4_ was used as a competitive inhibitor of NIS (*n* = 6). Data are expressed as mean ± SD. ** *p* < 0.01, * *p* < 0.05 (by Student’s *t*-test).

To determine whether RAI accumulation and enhancement of expression was better restored after treatment with TKI (0.5 µg/mL) and EVs^TKI(S)^ (0.5 µg/mL) than after treatment with DMSO (control), an ^125^I uptake assay was performed. The TKI treatment slightly increased the accumulation of iodine in the ATC cells, but this increase was not significantly different from that of the control treatment. However, the EVs^TKI(S)^ treatment showed a significant difference (*p* < 0.01) compared with the control treatment regarding the increase in iodine accumulation in the ATC cells. Moreover, we examined the relationship between iodine uptake and NIS expression. We used KClO_4_, a well-known inhibitor of NIS, in this analysis. The increased iodine accumulation from treatment with EVs^TKI(S)^ was completely blocked by the KClO_4_ treatment (Figure 7). These results confirm that the delivery of TKI by EVs (EVs^TKI(S)^) increases the reinforcement of iodine uptake by means of NIS.

## 4. Discussion

Recent studies suggest that EVs are continually evolving as a good candidate for therapeutics or drug delivery beacons [28]. The number of National Institutes of Health-registered clinical trials investigating EV-based therapeutics continuously increases every year [13,14,29,30]. A recent study using EVs as a delivery vehicle for chemotherapeutics in lung cancer patients demonstrated that EVs are feasible and safe [15]. We utilized EVs as nano-transporter for drug delivery, as it is a necessity to find a method for efficient drug (TKI) loading. Two different approaches for loading drug into EVs (sonication and incubation) were investigated to identify the most efficient loading method. Furthermore, changes in the efficacy of TKI (reverting iodine avidity in radioactive iodine-refractory thyroid cancer) were assessed by loading it in EVs.

Despite intense research being conducted on the use of EVs in drug delivery, stem cell-derived EVs have only been studied recently [19,31,32]. Isolation of EVs from MSCs has drawbacks, such as the highly invasive and painful procedure requiring general or spinal anesthesia that is used in obtaining bone marrow-derived stem cells (BMSCs). Therefore, BMSCs are not readily available for research and translational studies [33,34,35]. However, ADSCs are considered to be a promising source, and they are a readily available source. Many adipose tissues are discarded following liposuction procedures or other surgeries, and there are 500-times more stem cells isolated from adipose tissue than from bone marrow aspirates [36,37,38]. Previous studies report that the proliferative ability of ADSCs is greater than that of BMSCs [39,40,41]. ADSC EVs for loading TKIs can be isolated from autologous ADSCs cells, and the procedure to do this is minimally invasive and may bypass any unwarranted immune responses.

First, we isolated stem cells from adipose tissues and characterized them by morphology and expression of cell surface markers. The isolated stem cells showed the typical stem cell morphology, a fibroblast-like morphology, and they were strongly positive for CD90 and CD105. These observations and results are in agreement with those from previously reported studies [42,43]. The EVs were isolated from ADSCs cultured media, and they were enriched without cell or cell organelle contamination, as confirmed by enrichment of the EV markers CD81 and ALIX and absence of the mitochondrial protein cytochrome c. Moreover, these EVs displayed the typical EV morphology of a spherical shape, indicating they were intact. The isolated EVs were in the size range of 50–200 nm. The EVs were secreted by ADSCs, and thereby they were cell-derived and enriched, with no cellular contamination. These observations are consistent with the findings from previous studies [24,44,45].

Even though the use of EVs as a nano-transporter to deliver therapeutic materials has been studied only recently, they are well-known for their properties of remaining in the circulation for long periods, escaping degradation, evading the immune system, and penetrating tissue [5,46]. However, the effectiveness of EV-based drug delivery therapies depends on the loading of a substantial amount of therapeutics (drugs) without affecting their integrity, as a targetable delivery of EVs to the target is the overarching goal. The aim of the current study was to effectively deliver the newly identified TKI, 5-(5-{4H,5H,6H-cyclopenta[b]thiophen-2-yl}-1,3,4-oxadiazol-2-yl)-1-methyl-1,2-dihydropyridin-2-one, by EVs in ATC cells and provide evidence of its ability to augment iodine uptake in the ATC cells. There are various methods of drug loading into EVs, such as incubation, sonication, electroporation, freeze-thaw, extrusion, and saponification [5]. In the current study, we performed two commonly used methods to load the drugs into EVs, sonication and incubation, due to their simplicity and because they do not require specialized equipment (electroporator) or chemicals (saponin). We used the readily available equipment (sonicator and shaking incubator) that most laboratories have. Before loading the drug (TKI), we confirmed that it could be detected by mass spectrometry.

The TKI was mixed with the EVs and loaded into them via sonication or incubation, as illustrated in Figure 3. TKI was detected in both the EVs^TKI(S)^ and EVs^TKI(I)^, and no drugs were detected in naïve EVs before the drug was loaded into them. Our results show that five-time more TKI was loaded by the sonication method than by the incubation method, and our results are in agreement with those from previously reported studies [47,48]. The incubation and sonication procedures did not affect the morphology of the EVs after they were loaded with the drug, which is also in agreement with the results from previous studies using the incubation method [16,47]. A previous study showed that the sonication procedure aggregated the EVs, but this was not observed in our study. This difference may be due to the difference in the cells from which the EVs were derived [47]. However, comparative studies analyzing these differences are required to validate this hypothesis. To deliver the loaded drugs into recipient cells, EVs must navigate through their plasma membrane. Our results suggest that the EVs were internalized into the human ATC cells, regardless of whether the drug was loaded into the EVs using the sonication or incubation method, which is a finding that is consistent with those of previous studies showing that the internalization of EVs into recipient cells was not affected by drug loading [16,18,19,49].

At present, the removal of tumorous thyroid glands (thyroidectomy) followed by RAI therapy is the standard therapeutic strategy for differentiated thyroid cancers. Thyroid-specific transcription factors, including NIS, are frequently diminished or lose their function in poorly differentiated cancers and ATCs [50,51,52]. Thyroid cancers that do not express or show a low level of expression of NIS cannot be treated with RAI. Many pharmacologic interventions have tried to enhance endogenous NIS expression in thyroid cancers with negligible NIS expression. TKIs have been applied as a way to increase NIS expression from RAI therapy, which has attempted to increase the survival rate of thyroid cancer patients. Although it has been successful to some extent, the fundamental obstacles of TKI are its weak bioavailability, high dose requirements, adverse side-effects, low therapeutic effect, and nonspecific targeting [8,9,53,54]. EVs have the ability to overcome these fundamental obstacles for delivering TKI therapies to cancers.

We evaluated the in vitro effect of free TKI and EVs^TKI(S)^ with an equivalent amount of TKIs to a human ATC cell line, SW1736. The EVs^TKI(S)^ treatment restored the *NIS*, *TSHR*, and PAX-8 expression levels in ATC cells more effectively compared with free TKI at the same dose. Moreover, the EVs^TKI(S)^ treatment restored the NIS, TSHR, and PAX-8 protein levels in ATC cells more effectively compared with free TKI at the same dose. Furthermore, EVs^TKI(S)^ treatment restored the radioiodine avidity of ATC cells. These results confirm that treatment via the delivery of TKI by EVs is more effective compared with free TKI treatment in SW1736 cells. We need to further verify the effectiveness of this therapeutic strategy in other thyroid cancer cells. Previous studies have identified that TKI restores NIS expression. However, our study focused on the delivery of TKI as an effective way to enhance the benefits of RAI therapy for radioactive iodine-refractory thyroid cancer. Further study of the delivery of TKIs by EVs in vivo is warranted.

The EVs based nano-transporter is emerging as one of the potential ways to transporting the cargoes (small drugs, biological cargoes such as proteins, miRNAs, mRNA, and siRNA) [5,55]. Researchers and clinicians in the field have encountered several challenges to ensure the quality and quantity of EVs based nano-transporter [56]. Though there are over a hundred EV-related clinical trials, very few studies are focused as delivering the cargoes including drugs [55,56,57,58,59]. The current study has showed the successful drug (TKI) loading into EVs with highest loading was achieved by sonication methods, may pave was for further studies with EVs as a nano-transporter in preclinical and possible in clinical. We have also showed the accurate drug measurement after encapsulation in EVs’ detectable up to as low as 1 ng/mL by LC/MS, which could be a potential measurement tool for accurate measurement of drugs before administration to patients to avoid any possible toxicity.

In summary, we have successfully demonstrated that EVs can be isolated and purified from human primary ADSCs. TKI was successfully loaded into EVs by sonication and incubation, which we confirmed by MS analysis. Treating ATC cells with TKI-loaded EVs increased thyroid-specific proteins and transcription factors more than treating them with free TKI at the same dose. Our findings demonstrate that TKI-loaded EV treatment is more effective than free-TKI treatment for restoring RAI uptake in ATC cells. These results suggest that TKI-loaded EVs can be used to convert RAI-refractory thyroid cancer into a RAI-sensitive thyroid cancer in the future.

## Data Availability

Data will be made available on a reasonable request.

## Supplementary Materials

The following are available online at https://www.mdpi.com/1999-4923/13/2/248/s1, Figure S1. Isolation of EVs from ADSCs. Created with BioRender.com, Figure S2. Extracted Ion Chromatogram of TKI and Internal Standard (IS). (A) Blank EV with IS. (B) Standard TKI with IS. (C) TKI with IS spiked in EVs, Figure S3. TKI Loading into EVs did not affect the morphology and internalization. (A) Morphologies of EVs^(I)^ and EVs^TKI(I)^ cells were confirmed by transmission electron microscopy (scale bar: 200 nm). (B) ATC cells (SW1736) were incubated with EVs^(I)^, EVs^(I)^/DiD, or EVs^TKI(I)^/DiD (scale bar: 20 µm). Table S1: Summary of Intra-day and Inter-day TKI Concentrations in the EVs with RSD and Accuracy Calculations.
