# Supplementary material for: Extracellular Vesicles Act as Nano-Transporters of Tyrosine Kinase Inhibitors to Revert Iodine Avidity in Thyroid Cancer
Source: Pharmaceutics. 2021 Feb 10;13(2):248. doi: 10.3390/pharmaceutics13020248 (PMC7916551; doi:10.3390/pharmaceutics13020248)
Supplement: Supplementary file 1 [file pharmaceutics-13-00248-s001.pdf]

pharmaceutics

Supplementary Materials: Extracellular Vesicles Act as Nano-Transporters of Tyrosine Kinase Inhibitors to Revert Iodine Avidity in Thyroid Cancer

Ramya Lakshmi Rajendran [1], Sanjita Paudel [2], Prakash Gangadaran [1,3], Ji Min Oh [1], Eun Jung Oh [4], Chae Moon Hong [5], Sangkyu Lee [2], Ho Yun Chung [3,4], Jaetae Lee [1,5] and Byeong-Cheol Ahn [1,3,5,*]

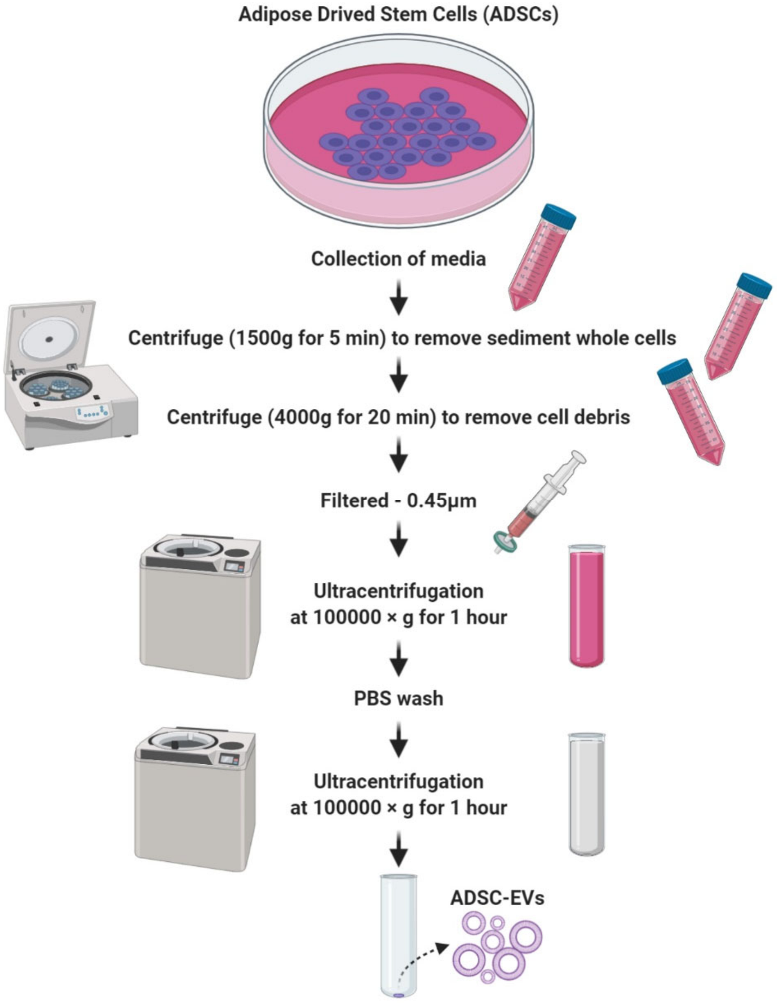

Figure S1. Isolation of EVs from ADSCs.

Publisher's Note: MDPI stays neutral with regard to jurisdictional claims in published maps and institutional affiliations.

Copyright: © 2021 by the author. Licensee MDPI, Basel, Switzerland. This article is an open access article distributed under the terms and conditions of the Creative Commons Attribution (CC BY) license (http://creativecommons.org/licenses/by/4.0/).

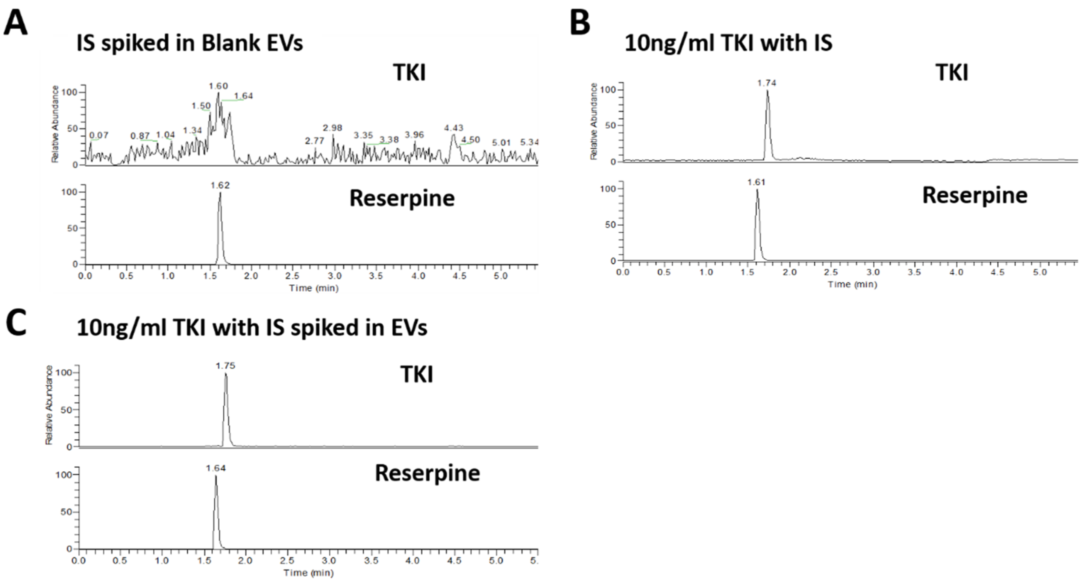

Figure S2. Extracted Ion Chromatogram of TKI and Internal Standard (IS). (**A**) Blank EV with IS. (**B**) Standard TKI with IS. (**C**) TKI with IS spiked in EVs.

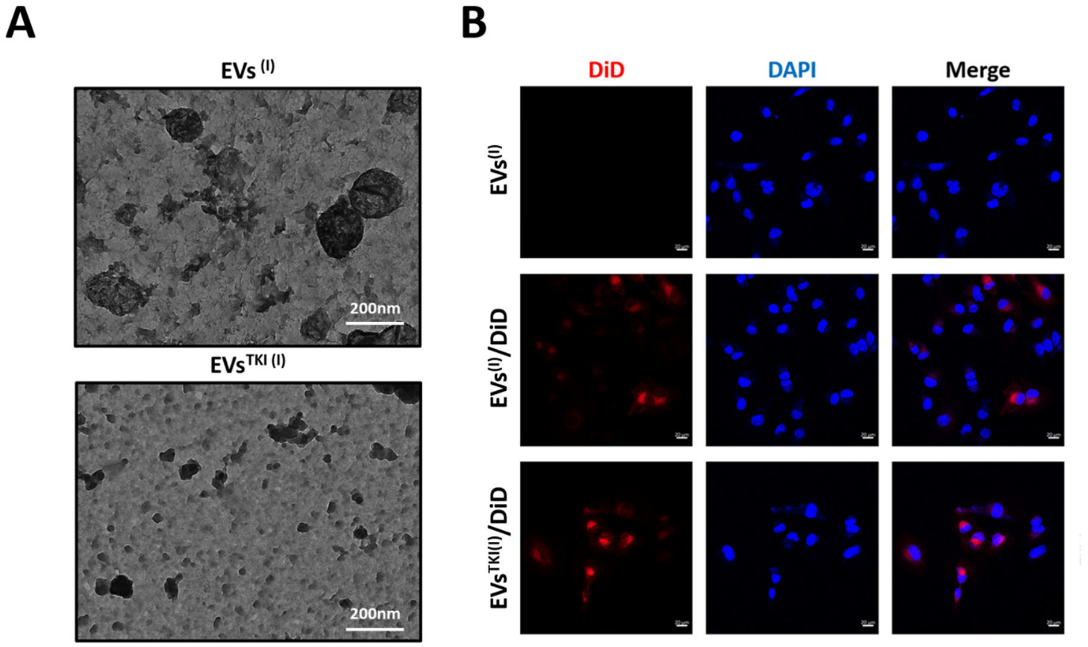

Figure S3. TKI Loading into EVs did not affect the morphology and internalization. (A) Morphologies of EVs$^{(I)}$ and EVsTKI$^{(I)}$ cells were confirmed by transmission electron microscopy (scale bar: 200 nm). (B) ATC cells (SW1736) were incubated with EVs$^{(I)}$, EVs$^{(I)}$/DiD, or EVs$^{TKI(I)}$/DiD (scale bar: 20 μm).

Table S1. Summary of Intra-day and Inter-day TKI Concentrations in the EVs with RSD and Accuracy Calculations.

Name of analyte	Nominal Concentration (ng/mL)	Intra-Day					Inter Day				
		Concentration Measured (ng/mL)			Precision (%, RSD)	Accuracy (%, RE)	Concentration Measured (ng/mL)			Precision (%, RSD)	Accuracy (%, RE)
**TKI**	**10**	**8.51**	**±**	**0.17**	4.7	114.8	8.70	±	0.20	5.2	112.9
	100	86.90	±	0.71	1.8	113.1	85.61	±	2.21	5.5	114.4
	1000	914.57	±	19.54	4.8	108.5	932.59	±	5.00	1.2	106.7
	5000	5049.34	±	115.42	5.1	99.0	5688.81	±	107.50	4.2	86.3
	20000	19612.29	±	192.31	2.2	101.9	21066.42	±	264.46	2.8	94.7
